# Using Smartwatches to Detect Face Touching

**DOI:** 10.3390/s21196528

**Published:** 2021-09-30

**Authors:** Chen Bai, Yu-Peng Chen, Adam Wolach, Lisa Anthony, Mamoun T. Mardini

**Affiliations:** 1 Department of Health Outcomes and Biomedical Informatics, College of Medicine, University of Florida, Gainesville, FL 32610, USA; malmardini@ufl.edu; 2 Department of Computer and Information Science and Engineering, University of Florida, Gainesville, FL 32611, USA; yupengchen@ufl.edu (Y.-P.C.); lanthony@cise.ufl.edu (L.A.); 3 Department of Aging and Geriatric Research, College of Medicine, University of Florida, Gainesville, FL 32610, USA; adam.wolach@ufl.edu

**Keywords:** smartwatch, accelerometer, face touching, machine learning, COVID-19, respiratory illnesses, wearables

## Abstract

Frequent spontaneous facial self-touches, predominantly during outbreaks, have the theoretical potential to be a mechanism of contracting and transmitting diseases. Despite the recent advent of vaccines, behavioral approaches remain an integral part of reducing the spread of COVID-19 and other respiratory illnesses. The aim of this study was to utilize the functionality and the spread of smartwatches to develop a smartwatch application to identify motion signatures that are mapped accurately to face touching. Participants (*n* = 10, five women, aged 20–83) performed 10 physical activities classified into face touching (FT) and non-face touching (NFT) categories in a standardized laboratory setting. We developed a smartwatch application on Samsung Galaxy Watch to collect raw accelerometer data from participants. Data features were extracted from consecutive non-overlapping windows varying from 2 to 16 s. We examined the performance of state-of-the-art machine learning methods on face-touching movement recognition (FT vs. NFT) and individual activity recognition (IAR): logistic regression, support vector machine, decision trees, and random forest. While all machine learning models were accurate in recognizing FT categories, logistic regression achieved the best performance across all metrics (accuracy: 0.93 ± 0.08, recall: 0.89 ± 0.16, precision: 0.93 ± 0.08, F1-score: 0.90 ± 0.11, AUC: 0.95 ± 0.07) at the window size of 5 s. IAR models resulted in lower performance, where the random forest classifier achieved the best performance across all metrics (accuracy: 0.70 ± 0.14, recall: 0.70 ± 0.14, precision: 0.70 ± 0.16, F1-score: 0.67 ± 0.15) at the window size of 9 s. In conclusion, wearable devices, powered by machine learning, are effective in detecting facial touches. This is highly significant during respiratory infection outbreaks as it has the potential to limit face touching as a transmission vector.

## 1. Introduction

Frequent facial self-touches, primarily during outbreaks, have the potential to be a mechanism of contracting and transmitting respiratory diseases such as the novel coronavirus (COVID-19). Despite the ongoing vaccination efforts during the COVID-19 pandemic to prevent further outbreaks, behavioral approaches (wearing masks, washing hands, social distancing, and reduced face touches) remain an integral part of reducing the spread of COVID-19 and other respiratory illnesses. According to the Centers for Disease Control and Prevention (CDC) [1], droplets coming from coughing or sneezing transmit many of the germs that cause respiratory illness. These germs usually spread through close contact with an infected person or through touching contaminated surfaces and then touching mucosal areas such as the mouth, nose, or eyes [2,3]. Detecting face touching using naturally worn devices such as smartwatches and providing real-time biofeedback to individuals would offer a highly scalable platform that has the potential to limit face touching and thus limit the spread of respiratory illness via fomite transmission. Biofeedback is a mind–body technique that helps in making individuals aware of their behaviors. Over time, they can learn to self-regulate their unconscious behavior without feedback [4]. The ability to alert individuals about their facial touches even after they occur is still beneficial to regulate these unconscious behaviors. 

According to a behavioral observational study conducted by Kwok and colleagues [5] on 26 students, people touch their faces, on average, 23 times per hour. About 44% of the time, the facial touch involves contact with mucosal areas such as the eye, nose, and mouth. Wearing a face mask, another commonly prescribed transmission reducing measure, reduced face touching but did not eliminate it. Those not wearing a mask touched their face spontaneously 20.1 times per hour, compared to 6.4 times per hour if wearing a mask [6]. This intrinsic behavior can lead to spreading the virus widely. One way to break the cycle and restrict the spread of the virus is to enhance the awareness of our unconscious face-touching activities—to alert individuals every time they touch their face. A similar intervention study has been conducted in the treatment of trichotillomania (hair pulling) [7]. This is viable through recognizing human movements using motion sensors (e.g., accelerometer) that are able to continuously capture body movements in high resolution. 

The International Data Corporation [8] showed that the shipments of wearable devices are expected to reach 637.1 million units in 2024 worldwide. In this study, we leveraged the popularity of smartwatches to collect and analyze tri-axial accelerometer data in a similar manner to a research-grade activity monitor, e.g., actigraph, to detect face-touching movements. Recent data from our own group demonstrated that the accelerometer hardware in a publicly available smartwatch yields a very similar output to a research-grade monitor [9]. Additionally, because smartwatches are purchased for personal use, researchers and practitioners could benefit from capturing data without needing to introduce new devices [10]. Unlike actigraphy devices being used today, this access provided the flexibility to establish a robust framework for personal data collection. New generations of smartwatches provide a standalone computing platform with access to 5G mobile data networks, powerful processors, large amounts of storage, and a diverse suite of physical sensors, which make them suitable for the goals of this study.

Despite the scarcity of published research aiming to recognize facial touches by leveraging wearable technology, there was a rise in the deployment of wearables during the COVID-19 pandemic [11]. Michelin et al. [12] utilized the inertia measurement unit (IMU) to collect tri-axial accelerometer and tri-axial gyroscope data from participants. They built a 1D convolutional neural network to detect face-touching movements. IMUs have also been utilized by other researchers to detect certain behaviors that involve the face such as eating [13,14] and smoking [15,16]. In our study, we focused on utilizing smartwatches to reach a large share of the population and build a scalable platform, unlike IMUs. Smartwatches have been utilized in a limited manner by researchers to detect face-touching movements. Sudharsan et al. [17] developed a smartwatch application to detect face-touching movements. Accelerometer data of face-touching activities were collected from four participants, and one-class classification models were trained to recognize face-touching activities. This effort demonstrated a proof of concept but was limited in both the number of activities and the number of participants which can affect the generalizability of the built models. 

The first step toward a face touching intervention involves validating a classification model capable of distinguishing movement signatures from in situ consumer wearable devices. In the present study, we developed a smartwatch application on the Samsung Galaxy Watch (Samsung, Seoul, South Korea) to collect raw accelerometer data. Data were collected while participants were performing the scripted activities in a laboratory setting, which is the first phase of the framework proposed by Keadle and colleagues [18] to evaluate devices that assess physical behavior (such as face touching). Then, we extracted data features from the raw accelerometer data and applied advanced machine learning techniques for face touching (FT) vs. non-face touching (NFT) recognition and individual activity recognition (IAR). The results are expected to provide a clear analytic approach to identifying motion signatures that are mapped accurately to face touching.

## 2. Materials and Methods

### 2.1. Participants

Participants were community-dwelling adults (20+ years old) who were able to read and speak English and were willing to undergo all testing procedures. Exclusion criteria included: failure or inability to provide informed consent; having cognitive impairment; being unable to communicate because of severe hearing loss or speech disorder; and having a medical condition that prevents the participant from performing the scripted activities. Ten participants (50% females, 47.7 ± 24.7 years old) were enrolled in the study. The goal of the study was to demonstrate the feasibility of the face touching detection approach, which explains the small number of participants. Additionally, this study was conducted during the COVID-19 pandemic, during which social restrictions were imposed that hindered the normal data collection process. The collected data from all participants were included in the analysis. The Institutional Review Board at the University of Florida approved all study procedures, and all participants provided written informed consent before the study. 

### 2.2. Study Procedure

Participants performed a battery of 10 activities at their own pace in a standardized laboratory setting. Activities were split into two categories: four face-touching (FT) activities that are deemed similar to or easy to confuse with FT; and six non-face-touching (NFT) activities that are relatively easy to be distinguished from FT. Activities are listed in Table 1 along with their description and category. Due to the lack of categorizing activities into FT and NFT in the literature, we chose these activities using our intuition by focusing on selecting face-touching activities that are common in our lives. Activities that are mainly performed at home, such as face washing, teeth brushing, and combing one’s hair, were excluded because they impose minimal risk. Our goal was to build an app and an analytic approach to protect individuals outside the home where the risk of coming into contact with fomites is higher. Participants were instructed to perform each task repeatedly for 3 min before taking a break and moving on to the next task. All the activities were completed in one visit in a randomly generated order to avoid any temporal dependence of behavior. 

### 2.3. Instrumentation

**Figure 1:**
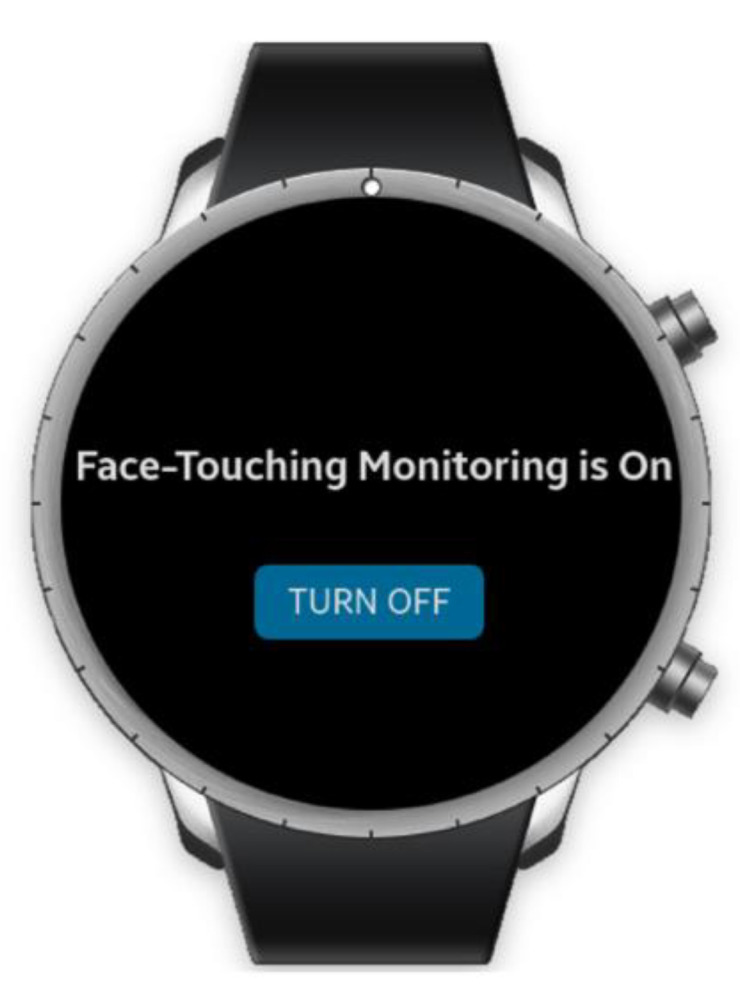
The smartwatch app user interface. The app collects continuous raw accelerometer data as long as it is on. The “TURN OFF” button allows the user to stop collecting data. The text above the button confirms that the data are being collected.

Participants wore a Samsung Galaxy smartwatch on their dominant wrist. Previous work suggests that wearing the accelerometer on the non-dominant or dominant wrist has no impact on physical activity assessment [19]. We opted to place the watch on the dominant wrist to standardize the data collection process and to make certain activities convenient for participants such as “writing”. The smartwatch is equipped with an embedded tri-axial accelerometer that records accelerations in units of gravity (1 g) in perpendicular, anterior–posterior, and medial–lateral axes. A predecessor to the watch, the Samsung Gear S, has a similar accelerometer that has been previously validated using a reciprocating shaker table [9]. We developed a smartwatch application (app) to collect raw acceleration data from the 3 axes at a sampling rate of 30 Hz. The user interface of the application is shown in Figure 1. The app begins collecting raw accelerometer data once the user opens the app. The text shown on the interface of the smartwatch indicates that the FT monitoring functionality is on and raw accelerometer data are being collected. The user can stop using the functionality and quit the application by pressing the TURN OFF button. This design has two-fold benefits: (1) it gives the user control over the app to only enable the FT functionality when needed; and (2) it reduces the amount of data collection and processing power to preserve the watch’s battery life. This design is aligned with the workout application on Apple and Samsung, in which the user turns on/off the app when necessary. Additionally, according to a study conducted on Samsung Gear S2 and S3 [20], collecting accelerometer data at 10 Hz for 2 h produced approximately 20 megabytes of data and depleted the battery by approximately 15–20%. Technically, detecting FT continuously throughout the day is not feasible at the moment. In addition to the smartwatch app, we developed a desktop application to record the start and end timestamps of each activity. To ensure the time alignment, the desktop application was synchronized with the smartwatch clock before each visit. The collected data were stored on the internal memory of the smartwatch. 

### 2.4. Problem Formulation and Data Processing

In this paper, we targeted two main tasks: (1) FT vs. NFT recognition (binary classification); and (2) IAR (multiclass classification). IAR helps in exploring the confusion among all activities and providing further insight into the predictive ability of each classifier. Before feeding the data into the machine learning algorithms, we applied preprocessing techniques. First, we eliminated the front-end and back-end noise by removing the first 20 and last 5 s of the accelerometer data for each activity, which has resulted in about 10 × 10 × (3 × 60 − 25) = 15,500 s of data. Second, we split the raw accelerometer data into smaller time segments (windows), as it is difficult to retrieve important and useful information from a continuous stream of sensor data [21]. Previous activity recognition studies used varying window lengths, ranging from 0.1 s to 128 s [22,23,24,25,26,27]. To select the best window size for FT recognition, we extracted features using consecutive non-overlapping windows with lengths ranging from 2 s to 16 s from the raw data. We did not take window lengths over 16 s into account, as the FT detecting application needs a quick response to provide feedback to users. Table S1 shows the average number of samples per participant and the total number of samples generated by the different window sizes. The selection of the window size was based on having sufficient data for accurate feature extraction and better accuracy. In total, 49 time- and frequency-domain features, listed in Table 2, were extracted [27,28]. 

### 2.5. Model Training

Machine learning models were developed for FT recognition and IAR. We examined four machine learning algorithms: logistic regression (LR), support vector machine (SVM), decision tree, and random forest, resulting in 8 models. For training and testing those models, we used nested cross-validation (nested-CV) with 10 outer folds and 3 inner folds. In each outer fold of the nested cross-validation process, a single participant served as a test set (leave-one-subject-out (LOSO)), and the other 9 participants served as a training set. Then, the outer training set was split again into three inner folds (three participants for each) in which each inner fold served as an independent validation set and the other two inner folds served as the inner training set (inner loop). In this way, for each outer fold, 6 participants were used to train the machine learning models, 3 participants served as a validation set, and the remaining participant was used as the test set. Figure S1 shows a graphical illustration of the process of nested cross-validation. Table 3 shows the numbers of samples in the training, validation, and test sets for minimum (2 s) and maximum (16 s) window lengths in each outer loop of the nested cross-validation. Considering the maximum window length (16 s), which will result in the smallest number of samples, each outer loop of the nested cross-validation will train the machine learning model using about 588 samples (data from 6 participants), validate (hyperparameter tuning) using about 294 samples (data from 3 participants), and finally test the performance of the model using about 98 samples (data from 1 participant). The inner loop is responsible for hyperparameter tuning (the process of searching for the optimal parameters of the model), while the outer loop is responsible for error estimation and generalization. In this approach, the model selection becomes an integral part of the model fitting process, which prevents bias in performance evaluation [29]. We used the grid search [30] for hyperparameter tuning, in which exhaustive combinations of the chosen hyperparameters were used for training the model. To overcome overfitting, we added the regularization term in logistic regression and support vector machine and we adopted pre-pruning by fine-tuning the maximum depths of the tree and the minimum number of samples required to be at a leaf node in the decision tree. Random forest, which is an ensemble of decision trees, is not prone to overfitting [31]. The final performance of the model was reported by averaging the performance of the outer folds. Accuracy, recall, precision, F1-score, and area under the curve (AUC) metrics were used to evaluate the performance of each machine learning method on the binary classification task. Accuracy, macro recall, macro precision, and macro F1-score were used for multiclass classification. For reproducibility purposes, we uploaded the code and detailed explanation of the algorithm to our GitHub repository [32].

## 3. Results

Table 4 shows the performance of the four machine learning algorithms on the FT/NFT recognition with a window length of 5 s. Logistic regression resulted in the best performance with a mean F1-score of 0.90. Decision tree resulted in the worst performance with a mean F1-score of 0.84.

Table 5 shows the performance of the four machine learning algorithms on the IAR with a window length of 9 s. Random forest demonstrated the best ability to recognize each individual activity, with the highest mean F1-score of 0.67. Meanwhile, decision tree resulted in the worst performance, with a mean F1-score of 0.56. 

**Figure 2:**
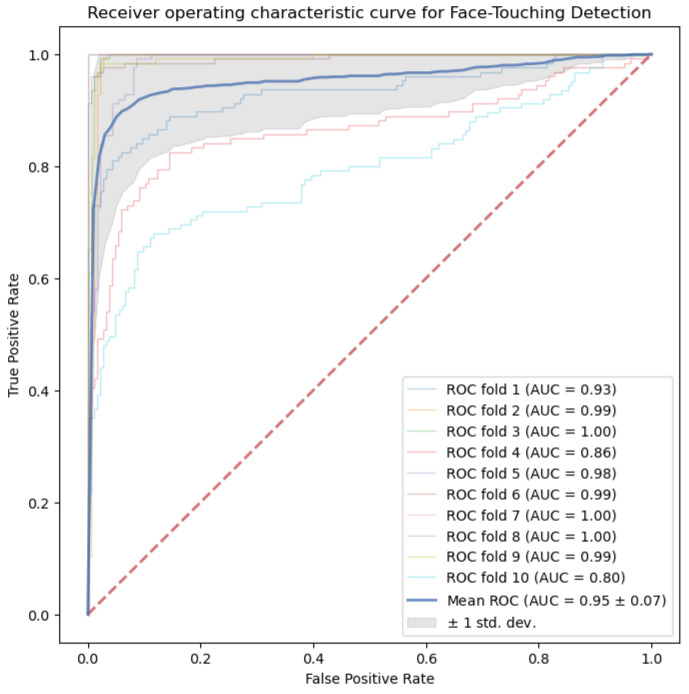
Receiver operating characteristic (ROC) curves for FT/NFT recognition from logistic regression.

Figure 2 shows the receiver operating characteristic (ROC) curve of logistic regression for the binary FT/NFT recognition task. The mean AUC of 0.95 obtained in our study indicates that the prediction performance of logistic regression on recognizing FT activity is relatively strong. Additionally, Figure S2 shows the training and testing accuracy of logistic regression for the FT/NFT recognition task. 

**Figure 3:**
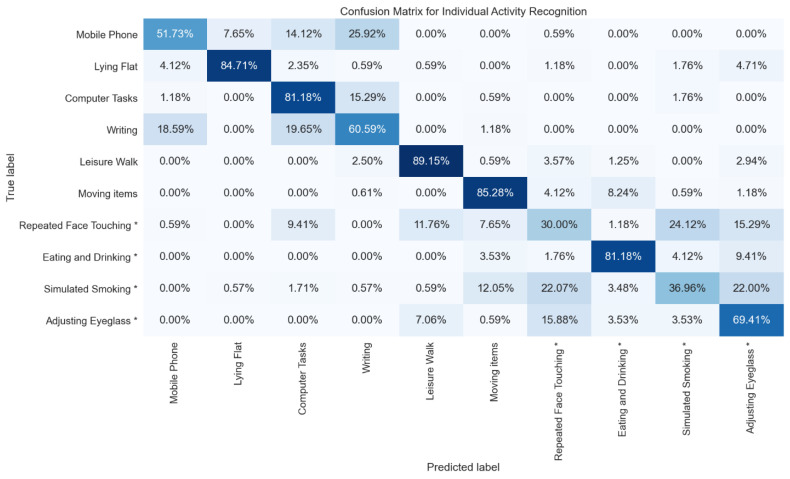
Confusion matrix of individual activity recognition; * indicates activities are FT activities.

Figure 3 shows the confusion between pairs of individual activities resulted from the random forest model. There are more intra-category confusions compared to inter-category confusions, which explains that recognizing FT/NFT models performed more optimally than IAR models. Figure S3 shows a randomly selected 9 s of a raw accelerometer signal for FT (repeated face touching) and NFT activities (leisure walk and moving items). Leisure walk signal is clearly distinguishable from repeated face touching. However, we observed some resemblance in movement patterns between moving items and repeated face touching.

Figures S4 and S5 show the top 15 features that contributed the most in the FT/NFT recognition task and the IAR task generated from the corresponding random forest classifier. It can be noticed that the ranking of features is relatively similar between the two tasks. The most important features in FT/NFT recognition and IAR are sdangle and min_x. In addition, the top five most important features are the same for both tasks.

Figures S6 and S7 show the accuracy of each machine learning method across different window lengths for FT/NFT recognition and IAR, respectively. Logistic regression achieved the highest accuracy among all four methods at the window length of 5 s in the FT/NFT recognition. Random forest achieved the highest accuracy among all four methods at the window length of 9 s in the IAR. 

Table S2 shows the comparison in performance of recognizing FT/NFT activities between the models built using the 2-s window (more samples) and the 16-s window (fewer samples). As can be noticed, the difference seems insignificant, despite the number of samples.

## 4. Discussion

### 4.1. Principle Results

The goal of this study was to examine the effectiveness of smartwatches and machine learning techniques to recognize FT motions, which is important during COVID-19 and other respiratory illness outbreaks. We analyzed raw accelerometer data collected from the wrist position. We utilized state-of-the-art machine learning algorithms: logistic regression, support vector machine, decision tree, and random forest for FT recognition and IAR. Results demonstrate that machine learning models were accurate at recognizing facial touches. However, models performed less optimally when recognizing individual physical activities. 

The highest F1-score for FT/NFT recognition was achieved by logistic regression with a mean F1-score of 0.90 followed by random forest (0.88), support vector machine (0.86), and decision tree (0.84). Although logistic regression only explores the linear relationship among features, it outperformed other complex classifiers that can explore non-linear relationships. These results are consistent with Zaki and colleagues [33], in which logistic regression outperformed other classifiers on two publicly available datasets of human activity recognition acquired from the UCI Machine Learning Repository [34]. 

Models built for IAR showed lower performance than recognizing FT/NFT. The overall deterioration in the performance of recognizing individual activity compared to recognizing FT activities is intuitive given the high number of classes. The highest F1-score for IAR was achieved by the random forest model, with a mean F1-score of 0.67. Summing these activities into categories such as the FT and NFT can help in enhancing the recognition performance metrics as observed in Table 4 and Table 5. Collapsing face-touching activities into one category in such a way can potentially reduce the risk of transmitting an infectious agent through direct or indirect touch (e.g., eating or adjusting glasses).

Figure 3 shows the confusion matrix of the IAR. The moving items activity from the NFT category has relatively large confusion (14.13%) with activities from the FT category. On the other hand, 12.05% of the simulated smoking activity was confused with the moving items activity. The moving items task consisted of three steps: (1) picking up a light item; (2) moving it to an adjacent spot; and (3) putting it down. The hand movement in this task is very similar to FT activities (lifting the hand toward the face, touching the face multiple times, and placing the hand down), except for a slightly longer duration needed to move the item to the adjacent spot. Therefore, the angle of the acceleration of the moving items task and FT tasks varied in a similar pattern. This angle is accounted for in the mangle and sdangle features extracted from the raw accelerometer data, which both ranked high in predicting FT motion as shown in Figures S4 and S5. A deeper look into the raw accelerometer data shows that the acceleration signals from leisure walk activity have a clear pattern, but the patterns of acceleration signals from the moving items and repeated face-touching activities are obscure as shown in Figure S3. 

The scaled impurity-based feature importance ranking generated from the random forest algorithm shows how relevant these features are to the problem at hand and helps in better understanding the model. We listed the top 15 features out of 49 features for both the FT/NFT recognition and IAR tasks. By examining the feature importance, there is a consistency in the ranking of these features across the two tasks. As elucidated earlier, the angle of acceleration plays an important role in recognizing FT activities. For example, the sdangle feature is ranked among the highest features in both tasks as shown in Figures S4 and S5. Being aware of the important features for the current task can help researchers continue improving model accuracy with less computational costs, which is highly significant in the case of real-time facial recognition on smartwatches. 

The selection of the length of the time window used to extract relevant features is a significant factor in our analysis. Smaller windows offer faster analysis and biofeedback but can lead to insufficient data for the problem at hand. Therefore, the selection should be based on a compromise between having sufficient data for accurate feature extraction and balancing computational resources. Our analysis showed that smaller window sizes are more appropriate in recognizing facial touches, unlike what is commonly used in the physical activity recognition literature [22,23,24,25,26,27]. The examined machine learning models for FT/NFT recognition achieved their highest performance at 3–9-s window lengths with 5-s being the best. The models for IAR achieved the best performance at the 9-s window length. However, the increase in the accuracy from 6- to 9-s window lengths was small (0.87%). In conclusion, smaller window lengths resulted in better performance for FT/NFT and IAR tasks.

Table S2 shows the comparison in performance between the models to recognize FT/NFT activities using the 2-s window and the 16-s window. The difference between the two models is insignificant, even though the model using the 2-s window has more samples. Recognizing FT/NFT seems to be a relatively simple task for different machine learning algorithms, where all models performed well and the difference was insignificant. However, the difference in performance was more obvious for the IAR task, as shown in Figure S7, across the different machine learning models because it is a more complex task. 

### 4.2. Comparison with Prior Work

There is a paucity of published research aiming to recognize facial touches by leveraging wearable technology. Generally, in this type of research, comparing relevant literature results is an intricate endeavor because of the differences in the data collection environment and the variables that govern the study. There are numerous differences between studies including sample size, the demographic characteristics of participants, the number and diversity of the physical activities tested, type of accelerometer, body position, statistical and machine learning algorithms applied, the extracted statistical features, the window size, and the metrics measured to evaluate the overall performance. However, some important comparisons can be made. Sudharsan and colleagues [17] developed a smartwatch app on the Samsung Gear S3 smartwatch to collect raw accelerometer data from four participants. They collected only hand-to-face movements (positive class) and applied one-class classification. Their machine learning models were built on data from three participants and tested on one participant who performed both FT and NFT activities. In the present study, data from ten participants of various ages performing multiple activities including FT and NFT movements were collected. This provides more generalizability and unbiased outcomes. Since our data are balanced, we used binary classification algorithms, but this does not imply a better approach. The ultimate choice of the classification paradigm depends on the problem at hand and data imbalance [35]. One major difference is that the authors processed and aggregated features from the whole time-series sequence of the FT movement rather than segmenting it into smaller time windows (epochs) as commonly approached in the literature. We expanded this work by examining the performance of different window sizes. Our analysis demonstrated that smaller window sizes (3–5 s) can result in high recognition performance, which is similar to the duration of the FT movement. However, we argue that segmenting the data into time windows is a safer option and allows the models to generalize better on unseen and potentially longer FT movements. Additionally, segmentation requires less computational power and provides faster analysis and biofeedback. Michelin et al. [12] proposed a face touching detection framework using a 1D convolutional neural network (CNN) with data from a tri-axial accelerometer and a tri-axial gyroscope. Their study included 40 participants and achieved a 92% accuracy using a window length of 500 ms. Despite the differences in our study, we achieved comparable performance by using a logistic regression model, which is simpler than the CNN and fits the computational and space limitations of smartwatches better. 

### 4.3. Limitations

A limitation of the current study is that data were collected in a controlled laboratory setting, though this is an appropriate first step in evaluating physical activity recognition methods [18]. Collecting data in free-living settings is more reflective of transitions between activities, but it is challenged by the need to label data. Other limitations include the small sample size, the small number of NFT activities involved, limited sedentary activities, and the limitation of detecting FT activities from one arm (the dominant wrist). The next step would involve evaluating the difference in performance of machine learning models on recognizing face-touching activities using data collected from the non-dominant and dominant wrist, increasing the number of participants and the number of activities, and building an algorithm to label FT movements in the individuals’ natural environment by considering the overlapping of activities simultaneously. Finally, we aim to examine the ability of shorter-time window sizes in detecting face touches. This will be the first step toward detecting face-touching movements when they are about to happen. 

## 5. Conclusions

In this study, we evaluated the effectiveness of machine learning approaches and wearable technology in recognizing face-touching movement patterns. Overall results suggest data features derived from wrist-worn accelerometers lead to high accuracy in recognizing face touches. This is highly significant during respiratory infection outbreaks as it has a great potential to inhibit individuals from touching their faces which is a transmission vector for respiratory illnesses. It also allows researchers and practitioners to utilize the publics’ personal devices to track and monitor their behavior in their natural environment and intervene when needed.

## Figures and Tables

**Table 1:** List of the performed activities and their category. Participants performed each one of the scripted activities for 3 min at their own pace.

Activity	Laboratory-Based Setting Description	Face Touching
Using mobile phone	Messaging using social media—no phone calls	No
Lying flat on the back	Simulating sleeping or napping	No
Computer tasks	Typing a document and navigating websites	No
Writing	Writing on a piece of paper	No
Leisure walk	Walking at a leisurely pace	No
Moving items from one location to another	Moving folding chairs from one location to another	No
Repeated face touching	Wiping nose, gestures on the face	Yes
Eating and drinking	Eating a snack and drinking water	Yes
Simulated smoking	Simulating the act of smoking	Yes
Adjusting eyeglass	Adjusting eyeglasses placed on the face	Yes

**Table 2:** Description of features extracted from the raw data.

	Feature	Description
Time	Mean of vector magnitude and acceleration from 3 axes (mvm, mean_x, mean_y, and mean_z)	Sample mean of VM, acceleration from x, y, and z axes in the window
	SD of vector magnitude and acceleration from 3 axes (sdvm, sd_x, sd_y, and sd_z)	Sample standard deviation of VM, acceleration from x, y, and z axes in the window
	Coefficient of variation of vector magnitude and acceleration from 3 axes (cv_vm, cv_x, cv_y, and cv_z)	Standard deviation of VM, acceleration from x, y, and z axes in the window divided by the mean, multiplied by 100
	Minimum value of vector magnitude and acceleration from 3 axes (min_vm, min_x, min_y, and min_z)	Minimum value of VM and acceleration from x, y, and z axes in the window
	Maximum value of vector magnitude (max_vm, max_x, max_y, and max_z)	Maximum value of VM and acceleration from x, y, and z axes in the window
	Twenty-five percent quantile of vector magnitude and acceleration from 3 axes (lower_vm_25, lower_x_25, lower_y_25, and lower_z_25)	Lower 25% quantile of VM and acceleration from x, y, and z axes in the window
	Seventy-five percent quantile of vector magnitude and acceleration from 3 axes (upper_vm_75, upper_x_75, upper_y_75, and upper_z_75)	Upper 75% quantile of VM and acceleration from x axis, y axis, and z axis in the window
	Third moment of vector magnitude and acceleration from 3 axes (third_vm, third_x, third_y, and third_z)	Third moment of VM and acceleration from x, y, and z axes in the window
	Fourth moment of vector magnitude and acceleration from 3 axes (fourth_vm, fourth_x, fourth_y, and fourth_z)	Fourth moment of VM and acceleration from x, y, and z axes in the window
	Skewness of vector magnitude and acceleration from 3 axes (skewness_vm, skewness_x, skewness_y, and skewness_z)	Skewness of VM, acceleration from x, y, and z axes in the window
	Kurtosis of vector magnitude and acceleration from 3 axes (kurtosis_vm, kurtosis_x, kurtosis_y, and kurtosis_z)	Kurtosis of VM, acceleration from x, y, and z axes in the window
	Mean angle of acceleration relative to vertical on the device (mangle)	Sample mean of the angle between x axis and VM in the window
	SD of the angle of acceleration relative to vertical on the device (sdangle)	Sample standard deviation of the angles in the window
Frequency	Percentage of the power of the vm that is in 0.6–2.5 Hz (p625)	Sum of moduli corresponding to frequency in this range divided by sum of moduli of all frequencies
	Dominant frequency of vm (df)	Frequency corresponding to the largest modulus
	Fraction of power in vm at dominant frequency (fpdf)	Modulus of the dominant frequency/sum of moduli at each frequency

**Table 3:** Number of samples in training, validation, and test sets for minimum and maximum window lengths in each outer loop of the nested cross-validation.

Window Size	Number of Samples in Training Set	Number of Samples in Validation Set	Number of Samples in Testing Set
2 s	4716	2358	787
16 s	588	294	98

**Table 4:** Performance metrics of recognizing face-touching activities (face touching vs. non-face touching). Each value is the mean and standard deviation of the 10-fold nested cross-validation.

Classifier	Accuracy	Recall	Precision	F1-Score	AUC
LR	0.93 (0.08)	0.89 (0.16)	0.93 (0.08)	0.90 (0.11)	0.95 (0.07)
SVM	0.89 (0.09)	0.85 (0.15)	0.89 (0.12)	0.86 (0.12)	0.92 (0.08)
Decision Tree	0.88 (0.09)	0.82 (0.18)	0.87 (0.10)	0.84 (0.13)	0.89 (0.10)
Random Forest	0.91 (0.10)	0.86 (0.17)	0.91 (0.12)	0.88 (0.14)	0.95 (0.08)

**Table 5:** Performance metrics for individual activity recognition. Each value is the mean and standard deviation of the 10-fold nested cross-validation.

Classifier	Accuracy	Recall	Precision	F1-Score
LR	0.65 (0.10)	0.66 (0.10)	0.65 (0.12)	0.62 (0.12)
SVM	0.66 (0.13)	0.66 (0.13)	0.66 (0.14)	0.64 (0.14)
Decision Tree	0.59 (0.11)	0.59 (0.11)	0.61 (0.13)	0.56 (0.12)
Random Forest	0.70 (0.14)	0.70 (0.14)	0.70 (0.16)	0.67 (0.15)

## Data Availability

Data and codes are available in our GitHub repository (https://github.com/ufdsat/FTCode).

## Supplementary Materials

The following are available online at https://www.mdpi.com/article/10.3390/s21196528/s1, Table S1: Average number of samples per participant and the total number of samples by window size from 2 to 16 s, Table S2: F1-score of recognizing face-touching activities (face touching vs. non-face touching) using the window size of 2 and 16 s for different classifiers. Each value is the mean and standard deviation of the 10-fold nested cross-validation, Figure S1: Illustration of how nested cross-validation works. Each block refers to one participant in the dataset, Figure S2: Training and testing accuracy of logistic regression for FT/NFT recognition task. Each pair of bars represents the accuracy for the corresponding outer loop of the nested cross-validation, Figure S3: Visualization of 9 s acceleration signal from leisure walk, moving items, and repeated face touching, Figure S4: Feature importance for face touching recognition, Figure S5: Feature importance for individual activity recognition, Figure S6: Accuracy of each machine learning method with different window lengths on FT/NFT recognition, Figure S7: Accuracy of each machine learning method with different window lengths on IAR.
