# Supplementary material for: Using Smartwatches to Detect Face Touching
Source: Sensors (Basel). 2021 Sep 30;21(19):6528. doi: 10.3390/s21196528 (PMC8513006; doi:10.3390/s21196528)
Supplement: Supplementary file 1 [file sensors-21-06528-s001.zip › sensors-1362295-supplementary.pdf]

Table S1. Average number of samples per participant and the total number of samples by window size from 2-16 seconds.

Window Size	Average number of samples per participant	Total number of samples
2	787	7868
3	523	5230
4	390	3900
**5**^{a}	**311**	**3107**
6	259	2593
7	220	2195
8	192	1920
**9**^{b}	**170**	**1697**
10	152	1521
11	139	1394
12	129	1293
13	119	1193
14	109	1094
15	100	998
16	99	987

a: the best window size for FT/NFT recognition

b: the best window size for IAR

Table S2. F1-score of recognizing face touching activities (face touching vs. non-face touching) using the window size of 2 and 16 seconds for different classifiers. Each value is the mean and standard deviation of the 10-fold nested cross-validation.

Classifier	2 seconds	16 seconds
LR	0.83 (0.11)	0.87 (0.13)
SVM	0.80 (0.15)	0.82 (0.22)
Decision Tree	0.82 (0.14)	0.80 (0.15)
Random Forest	0.84 (0.11)	0.86 (0.16)

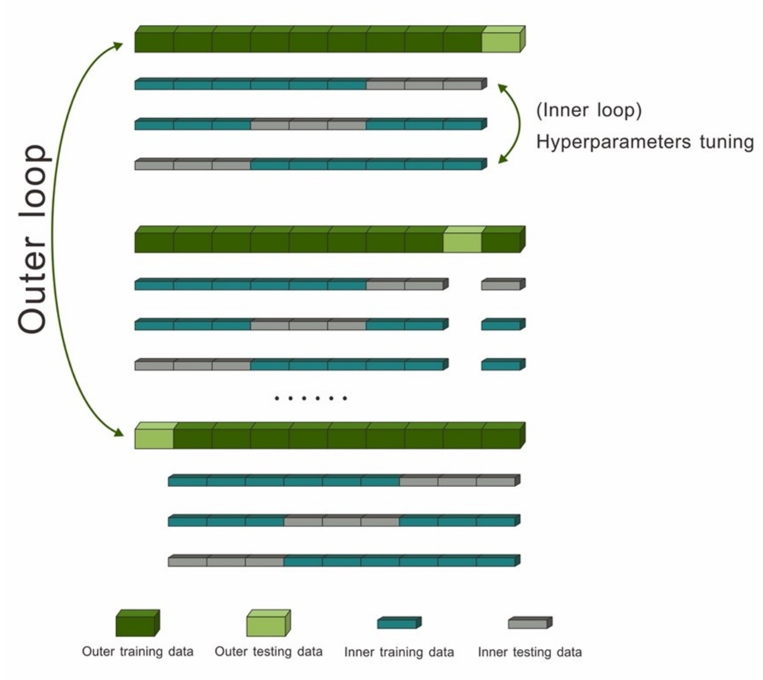

Figure S1. Illustration of how nested cross-validation works. Each block refers to one participant in the dataset.

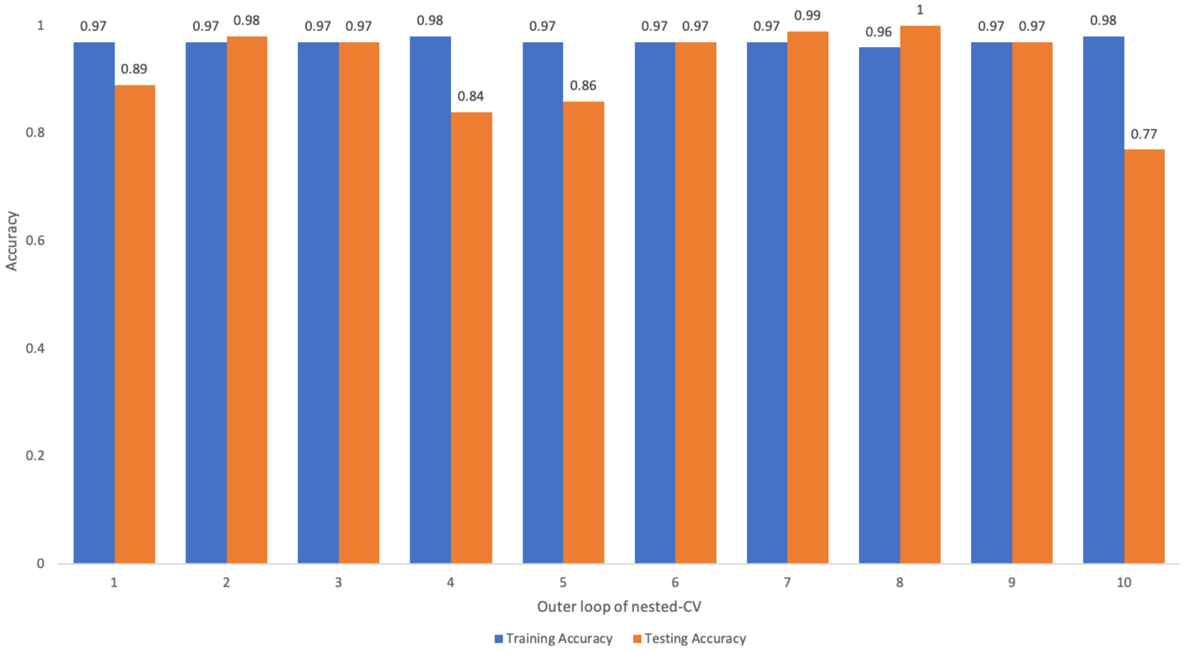

Figure S2. Training and testing accuracy of logistic regression for FT/NFT recognition task. Each pair of bars represents the accuracy for the corresponding outer loop of the nested cross-validation.

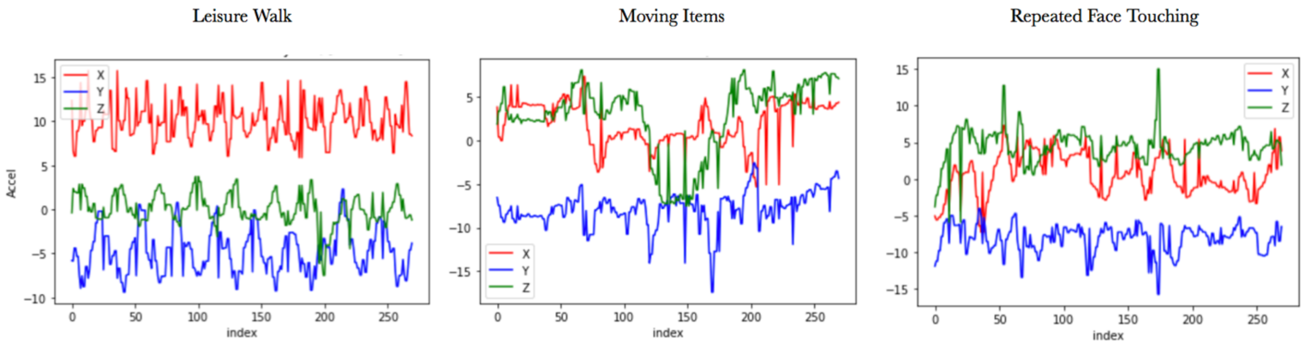

Figure S3. Visualization of 9s acceleration signal from leisure walk, moving items and repeated face touching.

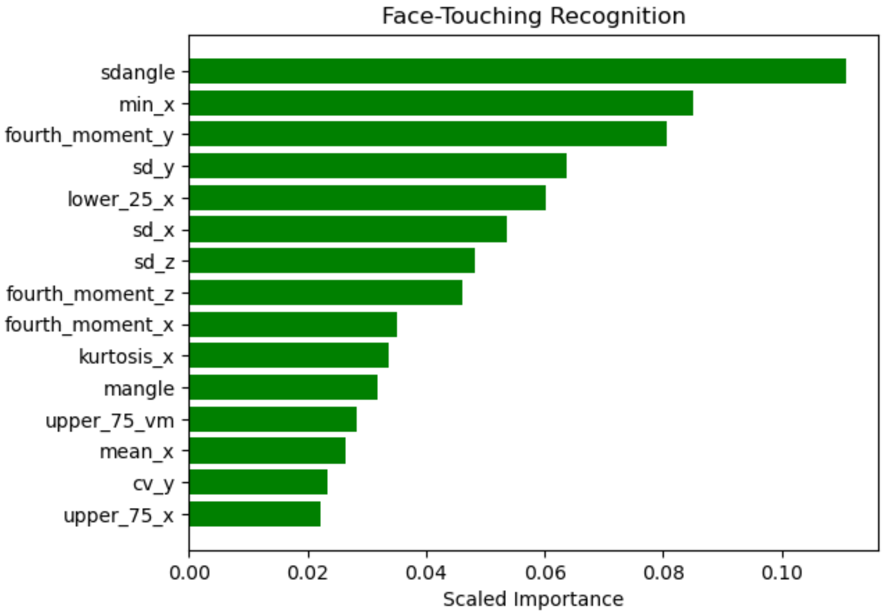

Figure S4. Feature Importance for Face-Touching Recognition.

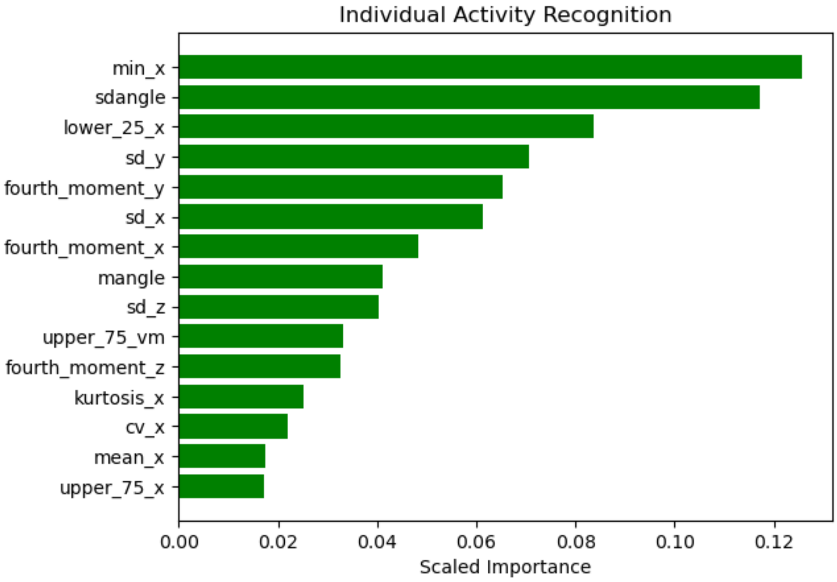

Figure S5. Feature Importance for Individual Activity Recognition.

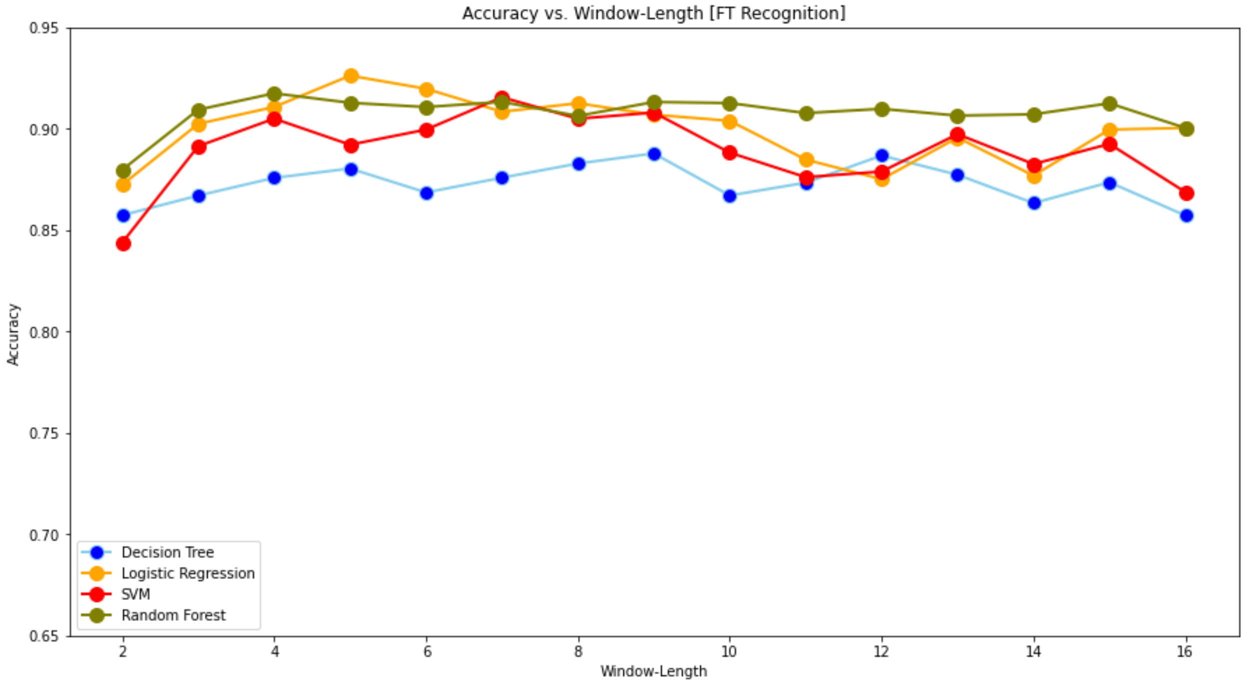

Figure S6. Accuracy of each machine learning method with different window lengths on FT/NFT recognition.

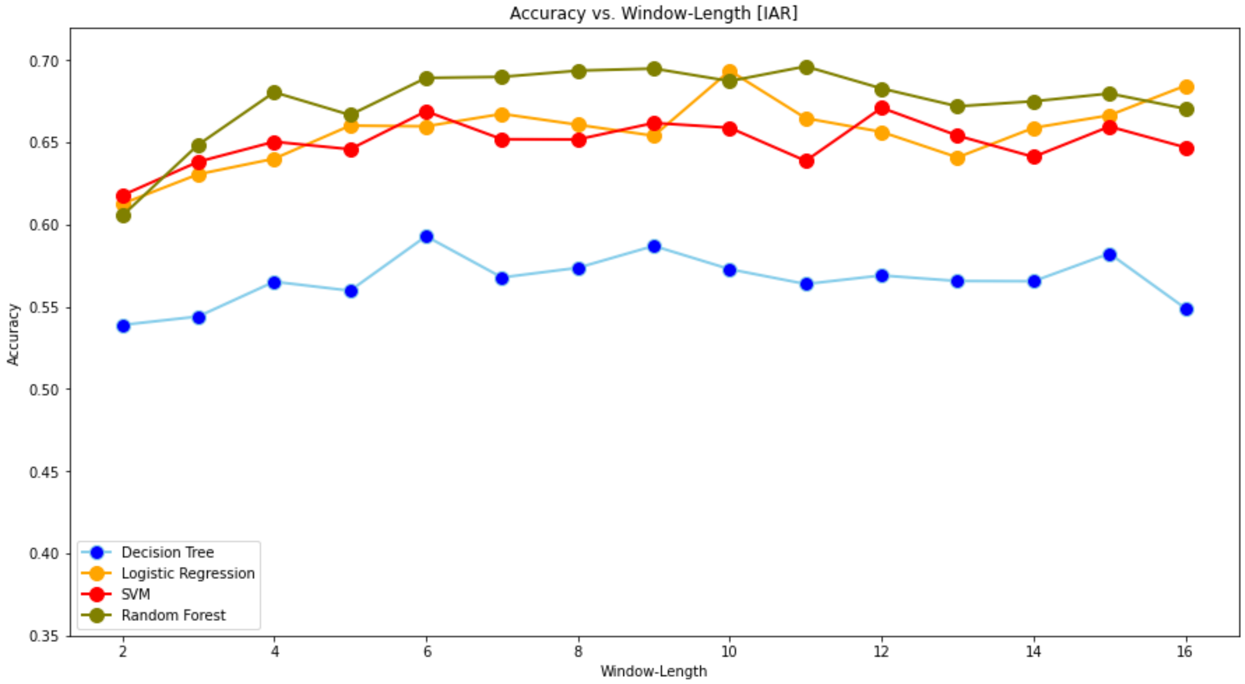

Figure S7. Accuracy of each machine learning method with different window lengths on IAR.
